# Highly efficient electroconversion of carbon dioxide into hydrocarbons by cathodized copper–organic frameworks†
†Electronic supplementary information (ESI) available. See DOI: 10.1039/c9sc02605c


**DOI:** 10.1039/c9sc02605c

**Published:** 2019-07-02

**Authors:** Fan Yang, Aling Chen, Pei Lin Deng, Yinzheng Zhou, Zaman Shahid, Hongfang Liu, Bao Yu Xia

**Affiliations:** a Key Laboratory of Material Chemistry for Energy Conversion and Storage (Ministry of Education) , Key Laboratory of Material Chemistry and Service Failure , School of Chemistry and Chemical Engineering , Wuhan National Laboratory for Optoelectronics , Huazhong University of Science and Technology (HUST) , 1037 Luoyu Road , Wuhan 430074 , PR China . Email: byxia@hust.edu.cn

## Abstract

Highly selective conversion of carbon dioxide (CO_2_) into valuable hydrocarbons is promising yet challenging in developing effective electrocatalysts. Herein, Cu^II^/adeninato/carboxylato metal–biomolecule frameworks (Cu^II^/ade-MOFs) are employed for efficient CO_2_ electro-conversion towards hydrocarbon generation. The cathodized Cu^II^/ade-MOF nanosheets demonstrate excellent catalytic performance for CO_2_ conversion into valuable hydrocarbons with a total hydrocarbon faradaic efficiency (FE) of over 73%. Ethylene (C_2_H_4_) is produced with a maximum FE of 45% and a current density of 8.5 mA cm^–2^ at –1.4 V *vs.* RHE, while methane (CH_4_) is produced with a FE of 50% and current density of ∼15 mA cm^–2^ at –1.6 V *vs.* RHE. These investigations reveal that the reconstruction of cathodized Cu^II^/ade-MOFs and the formed Cu nanoparticles functionalized by nitrogen-containing ligands contribute to the excellent CO_2_ conversion performance. Furthermore, this work would provide valuable insights and opportunities for the rational design of Cu-based MOF catalysts for highly efficient conversion of CO_2_ towards hydrocarbon generation.

## Abstract 2

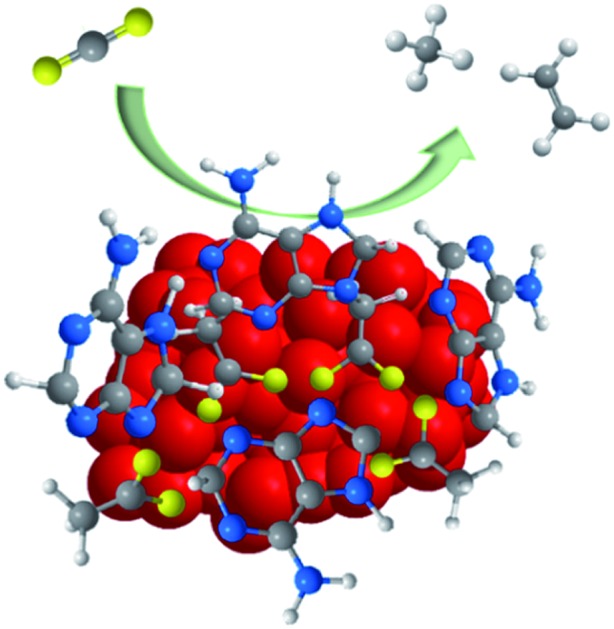
Cathodized Cu-MOFs (Cu–ade MOFs) exhibit structural evolution and contribute to efficient electrochemical CO_2_ reduction towards hydrocarbon generation.

## 

The rapid growth of global population and industrial development require high consumption of energy and fuels that leads to significant emission and accumulation of carbon dioxide (CO_2_), one of the main reasons for severe global warming and climate change.^1^,^2^ Natural photosynthesis is the only means to balance the CO_2_ level, however, it has limited capability in CO_2_ utilization and conversion to maintain the crucial carbon cycle.^3^ Therefore, developing efficient artificial CO_2_ fixation technologies are imperative to balance the carbon footprint and reduce excessive CO_2_ emissions for a sustainable human society.^4^ Except for the physical storage, chemical conversion of CO_2_ to valuable chemicals has been investigated extensively.^5^ Contrary to the thermochemical and photochemical approaches, electrochemical reduction of CO_2_ powered by renewable energy sources (solar, wind, *etc.*) is a useful possibility due to its mild reaction conditions/systems and various valuable products.^6^,^7^ Extensive research studies reveal that carbon monoxide (CO) and formic acid (HCOOH) are the main products of electrochemical CO_2_ conversion.^8^–^10^ However, high energy density and value-added hydrocarbons, including methane (CH_4_) and ethylene (C_2_H_4_) are more desirable for further industrial practices.^11^,^12^ Copper (Cu) based nanomaterials show the potential capability to generate various hydrocarbons.^13^–^16^ Nevertheless, these inorganic Cu compounds suffer from low efficiency and selectivity for CO_2_ conversion towards hydrocarbon production.^17^–^20^

Recently, porous metal–organic frameworks (MOFs) constructed from the coordination bonds of organic ligands and metal ions have attracted extensive interest.^21^ The adjustable molecular structures and metal categories make MOFs emerging alternatives for multidisciplinary catalysis applications.^22^,^23^ Especially, the atomic level periodicity of Cu complexes allows the design of Cu active sites for potential CO_2_ electrolysis.^24^–^26^ For example, Cu porphyrin molecules can convert CO_2_ to CH_4_ with a faradaic efficiency (FE) of ∼30% at –0.98 V *vs.* RHE,^23^ while Cu phthalocyanine shows an FE for CH_4_ of 66% at –1.06 V (*vs.* RHE).^25^ Nevertheless, crystalline Cu-MOFs themselves show a limited efficiency for electrochemical conversion of CO_2_ to C_2_ products. For example, the Cu(ii) benzene-1,3,5-tricarboxylate (btc) MOF (HKUST-1) facilitates the production of CH_4_ with a maximum faradaic efficiency of 27% and a partial current density of 4.4 mA cm^–2^ at –1.16 V (*vs.* RHE),^25^ while the Cu_2_(CuTCPP) MOF facilitates the formation of formate and acetate with a total current density of 4.5 mA cm^–2^ and total faradaic efficiency of 85%.^26^ Another type of Cu MOF, named [Cu_2_(ade)_2_(CH_3_COO)_2_], facilitates the formation of a little amount of CH_3_OH with 0.7% FE and C_2_H_5_OH with 0.5% FE for CO_2_ conversion at –1.55 V *vs.* RHE.^27^ Moreover, these Cu-based active materials often exhibit structural evolution under the cathodic reductive environment but are rarely reported.^25^,^28^ Therefore, developing suitable Cu-MOF nanocatalysts and investigating their structural evolution would be significant for further understanding MOF-based catalysts in highly selective CO_2_ conversion towards valuable hydrocarbon chemical production.

In this work, Cu–ade MOFs are employed to investigate their structure and performance for the selective conversion of CO_2_. Interestingly, the Cu–ade nanosheets demonstrate an excellent catalytic performance towards hydrocarbon production with a total hydrocarbon faradaic efficiency (FE) of over 73%. Predominantly, ethylene (C_2_H_4_) with a maximum FE of 45% is mainly produced at –1.4 V *vs.* RHE with a current density of 8.5 mA cm^–2^, while methane (CH_4_) is mainly produced at –1.6 V *vs.* RHE with an FE of 50% and a current density of ∼15 mA cm^–2^. Characterization results and electrochemical analysis reveal the reconstruction of cathodized Cu-MOFs accompanied by the formation of Cu nanocrystals. Along with the residual ligands, these active Cu crystals would be responsible for the excellent CO_2_ conversion towards hydrocarbon production. The contemporary approach towards structural evolution of Cu-MOFs for CO_2_ conversion to hydrocarbons would provide valuable understanding in developing efficient Cu-MOF catalysts for selective CO_2_ electrolysis and beyond.

The Cu–ade MOF nanosheets are constructed by coordinating Cu^2+^ ions with adenine and acetic acid (Fig. 1a).^29^–^31^ The biomolecular adeninato/carboxylato ligands not only scaffold the abundant pores of Cu–ade MOFs but may also contribute protons to enhance the CO_2_ electroreduction process due to the presence of N-containing functional groups.^32^–^35^ Three Cu–ade MOFs with different thicknesses, named Cu–ade nanosheets (s-Cu–ade), nanoplates (p-Cu–ade) and nanocuboids (c-Cu–ade), are prepared by tuning the volume ratio of methanol and water (Fig. S1†). X-ray powder diffraction (XRD) patterns of MOF products are consistent with metal–adeninate frameworks, which reveals the successful formation of crystalline Cu–ade MOFs (Fig. 1b and S1†).^36^ Scanning electron microscopy (SEM) demonstrates that the Cu–ade MOFs have a width of ∼500 nm and a length of several micrometers (Fig. 1c). Notably, the transparent property suggests the thin nanosheet structure of Cu–ade MOFs. The transmission electron microscopy (TEM) image further confirms the thin two-dimensional structure, and the transparent Cu–ade MOF nanosheet shows a porous structure with a smooth surface (Fig. 1d).

**Fig. 1:**
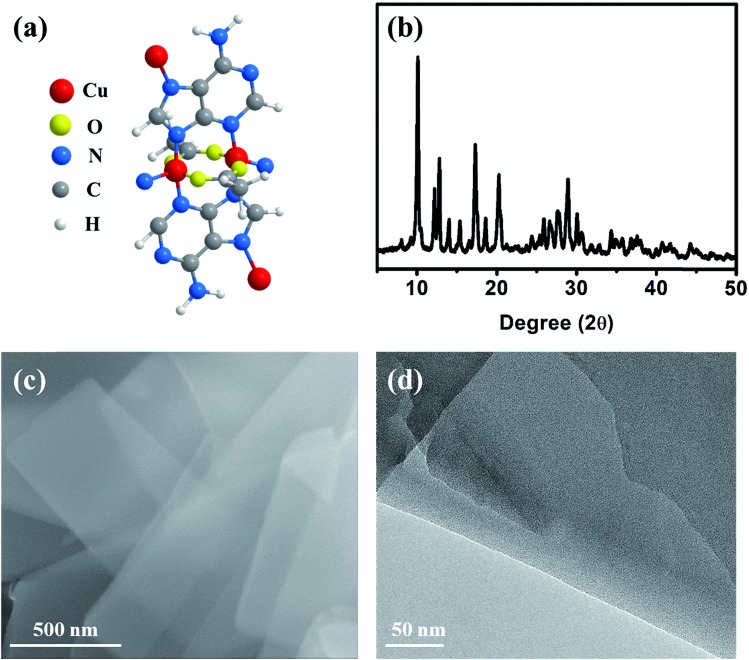
(a) Molecular structure of the Cu–ade monomer, (b) XRD pattern, (c) SEM image, and (d) TEM image of the s-Cu–ade MOF.

The CO_2_ electroreduction measurements of Cu–ade MOFs are performed in CO_2_-saturated 0.1 M KHCO_3_ electrolyte at the potential ranging from –1.2 V to –1.6 V *vs.* RHE. Fig. 2a demonstrates the potential-dependent current response in Ar-/CO_2_-saturated KHCO_3_ solution. Compared with the current response in Ar-saturated electrolyte, the significant enhancement of current density in the CO_2_-saturated electrolyte implies that the Cu–ade MOF catalysts may have potential catalytic activity for CO_2_ reduction. The gaseous products are monitored by using two online gas chromatography (GC) systems (Fig. S2†), and the liquid products are analyzed using the nuclear magnetic resonance (NMR) technique (Fig. S3†). When the Cu–ade catalysts are used in Ar-saturated 0.1 M KHCO_3_ solution, only the H_2_ product is detected without any carbon-containing gaseous and liquid products such as CO and CH_4_. However in the CO_2_-saturated 0.1 M KHCO_3_ solution, the major hydrocarbon products including CH_4_ and C_2_H_4_ are detected by GC measurements and few liquid products such as HCOOH and CH_3_CH_2_OH are characterized by NMR analysis (Fig. S3†). These results suggest that Cu-MOFs are effective for CO_2_ electrochemical reduction, whereas the reduction products are from CO_2_ rather than the carbon-containing Cu–ade catalyst system.

**Fig. 2:**
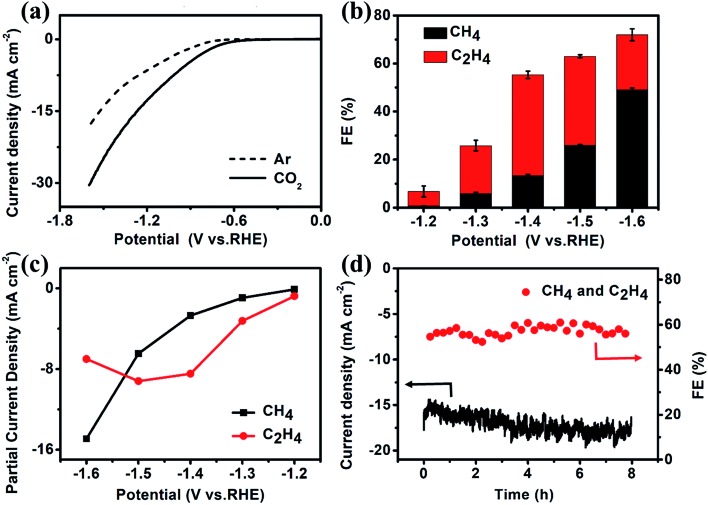
(a) LSV curves, (b) the FE of CH_4_ and C_2_H_4_, (c) partial current density of CH_4_ and C_2_H_4_, and (d) FE and current density test at –1.4 V *vs.* RHE for the s-Cu–ade MOF.

The constant potential test method is used to investigate the CO_2_ reduction over three Cu–ade MOF catalysts (Fig. S4†). The detailed analysis of CO_2_ electrochemical reduction shows that the main products of CO_2_ reduction are hydrocarbons and hydrogen (Fig. S5†). The faradaic efficiencies (FEs) of CH_4_ and C_2_H_4_ products over nanosheet structured Cu–ade MOFs are shown in Fig. 2b. C_2_H_4_ is first produced at a more positive potential than CH_4_, which means that the electrochemical reduction of CO_2_ preferentially generates the C_2_H_4_ product at a lower potential. The total FE of hydrocarbons (CH_4_ and C_2_H_4_) keeps increasing with more negative cathodic potentials and achieves the highest value of over 73% at a potential of –1.6 V *vs.* RHE. Among them, the FE of C_2_H_4_ increases firstly and then decreases while the FE of CH_4_ keeps increasing along with the increasing cathodic potentials. The maximum FE of C_2_H_4_ can reach 45% at –1.4 V *vs.* RHE, and the maximum FE of CH_4_ is over 50% at –1.6 V *vs.* RHE. This phenomenon is similar to other Cu-based electrocatalysts for hydrocarbon production by CO_2_ electrolysis, as larger potentials induce more difficult C–C coupling, thus resulting in decreased C2 products and increased C1 products.^37^–^39^ The partial current densities of CH_4_ and C_2_H_4_ at different potentials are shown in Fig. 2c. The partial current density of C_2_H_4_ is 9.2 mA cm^–2^ at –1.5 V *vs.* RHE, and the high current density is rare for MOF-derived catalysts for converting CO_2_ to C_2_H_4_.^23^,^25^–^27^ At a potential of –1.6 V *vs.* RHE, the partial current density of C_2_H_4_ is 15.0 mA cm^–2^ accompanied by a total FE of 73% for hydrocarbons (CH_4_ and C_2_H_4_). Compared to the reported Cu-complexes, these cathodized Cu-MOFs show excellent efficiency for the electrochemical conversion of CO_2_ to C_2_ products (Table S1†).

The electrolysis stability of Cu–ade MOF nanosheet catalysts is further investigated at –1.4 V *vs.* RHE for 8 hours (Fig. 2d). The total FE of CH_4_ and C_2_H_4_ is stable at ∼60% during the whole stability test. However, the total current density increases slightly, which might be due to some side reactions, such as hydrogen evolution and the gradual reduction of Cu–ade MOF nanosheets. The detailed FE evolution of CH_4_ and C_2_H_4_ is demonstrated in Fig. S7d.† With prolonged electrolysis, the FE of the C_2_H_4_ product decreases and the FE of CH_4_ increases as time goes on. A similar trend is observed when electrolysis is performed at relatively negative electrode potentials; the FE of C_2_H_4_ decreases, and the FE of CH_4_ increases.

Among the three Cu–ade MOFs, s-Cu–ade MOF nanosheets demonstrate the most significant partial current density and the highest FE for hydrocarbons. However, linear scan curves of CO_2_ reduction over the three Cu–ade MOFs are similar (Fig. 3a). Very interestingly, the LSV curve of the s-Cu–ade in the Ar-saturated electrolyte is in the middle, which implies that the hydrogen evolution side reaction over the s-Cu–ade MOF may be inhibited. The lower FE of H_2_ also indicates this phenomenon (Fig. S5†). The FE of both CH_4_ and C_2_H_4_ is more than 50% at –1.4 V *vs.* RHE (Fig. 3b), and the partial hydrocarbon (CH_4_ and C_2_H_4_) current density of s-Cu–ade is also higher at a relatively negative potential (Fig. 3c). The other two Cu–ade materials show the same change rules in the FE of CH_4_ and C_2_H_4_. The FE of C_2_H_4_ first increases to ∼35% at –1.4 V *vs.* RHE and then declines, and the FE of CH_4_ always increases with the increased potentials (Fig. S6 and S7†). Among the three Cu–ade MOFs, the performance of CO_2_ reduction to the hydrocarbons (CH_4_ and C_2_H_4_) is dependent on the thickness of Cu–ade MOFs, and the s-Cu–ade nanosheets demonstrate the best CO_2_ reduction performance (Fig. 3 and S5†). Considering the same molecular structure and similar CO_2_ reduction behaviors in the activity and selectivity (Fig. S7†), the electrochemically active surface area (ECSA) and electrochemical impedance spectroscopy (EIS) measurements are further performed. To a certain extent, the ECSA represents the number of active sites determined by the electrochemical double layer capacitance (Fig. S8†). The capacitance is calculated by plotting current density differences against scan rates and the capacitance values of p-Cu–ade, c-Cu–ade and s-Cu–ade are ∼92 μF cm^–2^, ∼100 μF cm^–2^ and ∼138 μF cm^–2^, respectively, (Fig. 3d) and the roughness factors (*R*_f_) are 4.6, 5.0 and 6.9, respectively. Thus, ECSAs for p-Cu–ade, c-Cu–ade and s-Cu–ade are 0.9 cm^2^, 0.98 cm^2^, and 1.34 cm^2^, respectively. The area-based activities of three samples demonstrate a similar trend in the CO_2_ reduction activity (Fig. S9†), indicating that the enhanced CO_2_ reduction activity of Cu–ade MOF nanosheets is mainly ascribed to improved ECSAs, which provide more active sites that are exposed to the reactant.

**Fig. 3:**
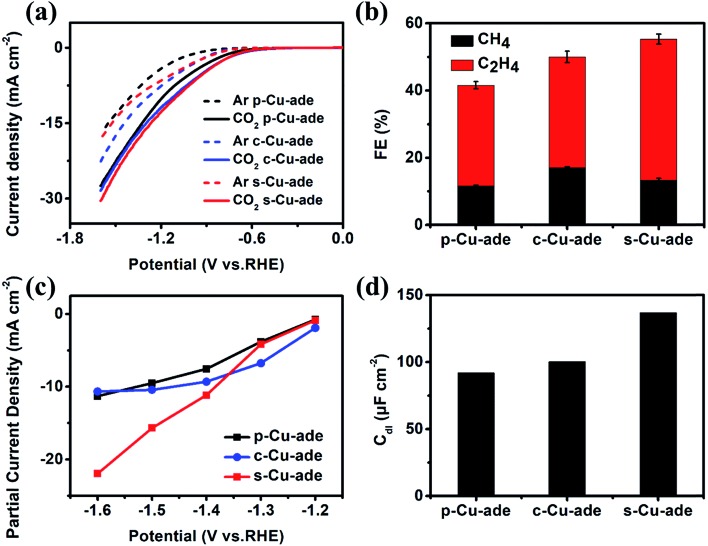
(a) LSV curves, (b) CH_4_ and C_2_H_4_ FE at –1.4 *vs.* RHE, (c) partial current density of hydrocarbons (CH_4_ and C_2_H_4_) and (d) *C*_dl_ comparison of the three Cu–ade MOFs.

Furthermore, the electrochemical impedance spectroscopy (EIS) measurement results and the equivalent circuit for the Nyquist plots are displayed in Fig. S9b.† The EIS data are fitted by the internal resistance (*R*_1_), the charge transfer resistance (*R*_s_), and the constant phase elements (CPE 1 and 2). Cu–ade MOF nanosheets exhibit the smallest transfer resistance (*R*_ct_), which indicates fast charge transfer during the electrochemical reaction process. The thin structure could contribute to sufficient contact of the reactant on nanosheets, which will result in fast charge transfer over the Cu–ade nanosheet catalyst to promote the hydrocarbon generation. Although the distribution of the reduction product mainly depends on the microstructure and intrinsic active Cu-complex sites, obviously, the different morphology of Cu–ade MOFs determines the activity of CO_2_ reduction. Moreover, the thinner nanosheets with more active sites exposed would lead to the enhanced activity for hydrocarbon generation.

The excellent performance in hydrocarbon generation attracts our attention to investigate the real active surface of Cu–ade MOFs. After the CO_2_ electrolysis, the morphology of Cu–ade MOFs demonstrates significant changes (Fig. 4 and S10†). SEM observations show that the morphology of initial Cu–ade MOF nanosheets is destroyed (Fig. 4a). Only the interconnected nanoparticles are present in the final cathodized MOFs. The corresponding TEM images of cathodized Cu–ade MOFs after the CO_2_RR also indicate nanoparticle aggregation (Fig. 4b inset). High-resolution (HR) TEM images show the inter-planar spacings of ∼0.18 nm and 0.21 nm, which are related to the Cu(200) and Cu(111) facets, respectively (Fig. 4b). The XRD pattern of Cu–ade MOFs after the electrolysis also demonstrates that the crystalline structure is completely changed (Fig. 4c), due to the missing peaks for Cu–ade MOFs and the emergence of Cu crystals (JCPDS: 85-1326). The diffraction peaks located at 43.3°, 50.4° and 74.1° are attributed to Cu(111), Cu(200) and Cu(220), respectively (Fig. 4c), which is consistent with the electron microscopy observations (Fig. 4b). Obviously, metallic Cu nanoparticles are formed *in situ* by the reduction of Cu–ade MOFs during the CO_2_ electroreduction process. The other two Cu–ade MOFs also exhibit similar morphologies and structural evolution (Fig. S10†).

**Fig. 4:**
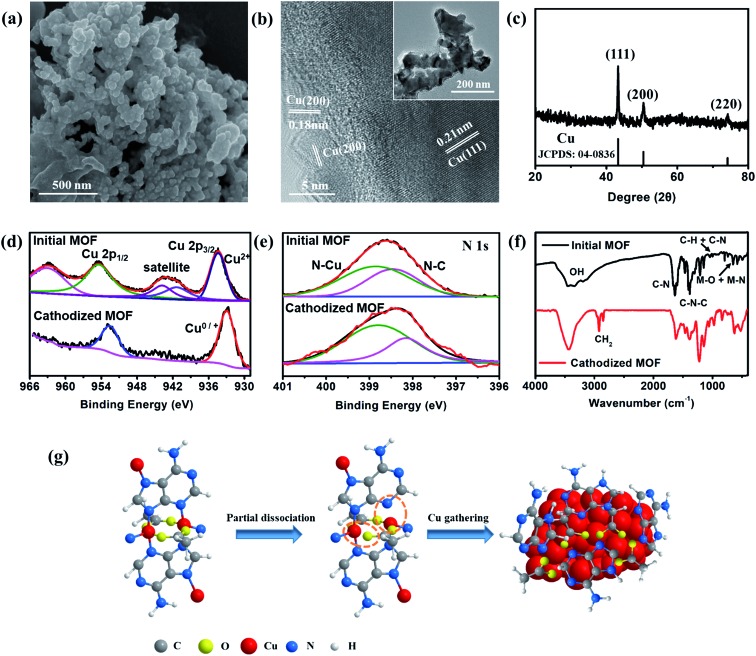
(a) SEM image, (b) TEM image, and (c) XRD pattern of the cathodized s-Cu–ade MOF. (d) Cu 2p and (e) N 1s scan XPS patterns and (f) FT-IR spectra of the initial and cathodized Cu–ade MOFs. (g) Proposed Cu–ade MOF evolution.

Elemental distribution indicates that the cathodized MOFs demonstrate two distinct element distributions, one is the copper enrichment region with Cu nanoparticles (Fig. S11a and b†), and another is the carbon enrichment region along with homogenous Cu element distribution (Fig. S11c and d†). For the Cu-rich area, it can be inferred that the Cu–ade coordination bond is broken from the Cu–ade MOF, and some Cu ion species are reduced to Cu clusters following the consequent segregation into copper nanoparticles. While for the C-rich area, it would be the organic ligands along with the residual Cu species. Nevertheless, the elemental mapping shows that other elements (C, O, and N) are uniformly distributed on the area of the entire cathodized sample including the Cu-rich area (Fig. S12†). The surface of Cu–ade MOFs is characterized using the XPS technique (Fig. S13†). The Cu 2p XPS spectra of initial Cu–ade MOFs confirm the presence of Cu(ii) species (Fig. 4d). After the cathodized CO_2_ electrolysis, the Cu(ii) satellite peaks disappear (Fig. 4d), while the Cu 2p_3/2_ and Cu 2p_1/2_ belonging to Cu(0)/Cu(i) appear at 932.5 and 952.3 eV (Fig. 4d). Further Cu LMM Auger peaks at ∼570.2 eV and ∼567.7 eV indicate the existence of Cu(i) and Cu(0) (Fig. S13†). Moreover, the C 1s and O 1s XPS patterns of the initial and cathodized s-Cu–ade MOFs show that the ratio of the C–N peak decreases and the O–Cu peak disappears, which indicates the formation of metallic Cu in the cathodized Cu–ade MOF nanosheets. The coordination environment of N species was analyzed using the XPS results (Fig. 4e). In the initial Cu-MOFs, N binds with C and Cu to form stable MOFs (Fig. 4e). After the electrolysis, the N–C binding demonstrates a slight decrease, while the N–Cu binding increases accordingly (Fig. 4e). This transformation suggests the splitting of N–C ligands and the enhancement of N-containing ligands on the resultant Cu nanocrystals, which agrees well with the similar structural evolution in the microscopy observation (Fig. S12†). Moreover, the Fourier-transform infrared spectroscopy (FTIR) pattern also indicates the MOF structure evolution and the residual N–Cu and N–C bonds (Fig. 4f).^31^ The peak at ∼2900 cm^–1^ is related to H–C–H, and the enhanced peak at ∼550 cm^–1^ suggests the partial broken N–C and formed N–C in the cathodized Cu–ade MOF. Besides, the CuO nanosheet derived from s-Cu–ade is used to catalyze CO_2_ reduction. As observed from the SEM and XRD results (Fig. S14†), the surface of inorganic CuO becomes rough after annealing Cu-MOFs. The primary product of CO_2_ reduction is CH_4_ at a relatively negative potential. The maximum FE of CH_4_ reaches 32% at –1.6 V *vs.* RHE. Therefore, this result, to a certain extent, proves that the as-formed Cu functionalized by the nitrogen containing organic ligands in the cathodized Cu-MOFs is essential for converting CO_2_ toward hydrocarbon generation.

CH_4_ and C_2_H_4_ are the major products of cathodized Cu-MOF catalyzed CO_2_ reduction. In the cathodized MOFs, the derived Cu nanoparticles demonstrate the formation of Cu clusters with abundant (111) and (100) facets. The low-index Cu(100) plane favors the production of C_2_H_4_ while the Cu(111) facets are mainly responsible for the CH_4_ yield.^40^ Moreover, from the full potential FE of C_2_H_4_ and CH_4_ (Fig. 2b) and the stability test (Fig. S7d†), the FE of C_2_H_4_ product decreases and the FE of CH_4_ increases with enhancing the potential or prolonging the electrolysis time. It is evident that the formation of CH_4_ and C_2_H_4_ is competitive in the reaction kinetics to some extent, the pathway of C_2_H_4_ would be inhibited, and then the generation of CH_4_ could be promoted along with the increased potential or electrolysis time. The CO* dimerization pathway is sluggish, yet this can't be omitted from the reaction pathway for C2 (C_2_H_4_) species under highly negative potentials,^41^ consequently, the reaction for CH_4_ and C_2_H_4_ products undergo the hydrogenation and dimerization of the intermediate (COH* or CHO*).^41^ After the formation of the CO* intermediate, the COH* or CHO* is the next critical intermediate for further hydrogenation to form CH_4_ or dimerization to produce C2 species on Cu(111) and Cu(100).^42^,^43^ Compared to the formation of C_2_H_4_ by the dimerization process with a higher barrier, the intermediates (COH* and CHO*) prefer to couple with protons and undergo hydrogenation to form CH_4_. Furthermore, the N 1s XPS spectra show the decreased N–C proportion and enhanced N–Cu in the cathodized Cu-MOFs (Fig. 4 and S13†). The presence of N-containing groups would activate the protons and stabilize the intermediates CO*/CHO* of CO_2_ reduction to promote further hydrogenation to form hydrocarbons.^32^ Therefore, the higher ratio of N–Cu in the cathodized Cu-MOFs would probably determine the efficient electrochemical reduction of CO_2_ towards hydrocarbons. Based on the experimental observation and analysis results, the CO_2_ reduction process over the cathodized Cu-MOFs could be proposed. Under the reductive environment, the bond between Cu and ade ligands could be broken and the organic N-containing ligands mostly cover the formed Cu nanoparticles. Benefitting from the presence of N–Cu, the hydrogenation and dimerization of CHO*/COH* could more easily occur to form hydrocarbons.

In summary, cathodized Cu–ade-MOFs have been investigated for efficient electrochemical CO_2_ reduction towards hydrocarbon generation. The cathodized Cu^II^/ade-MOF nanosheets demonstrate excellent catalytic conversion towards hydrocarbon production with a total hydrocarbon faradaic efficiency (FE) of over 73%. Primarily, ethylene (C_2_H_4_) is mainly produced at –1.4 V *vs.* RHE with a maximum FE of 45% and a partial current density of 8.5 mA cm^–2^, while methane (CH_4_) is mainly produced at –1.6 V *vs.* RHE with a FE of 50% and partial current density of ∼15 mA cm^–2^. It is found that Cu–ade MOFs exhibit *in situ* structural evolution under the reductive CO_2_ environment. The reconstruction of cathodized Cu^II^/ade-MOFs and the formed Cu nanoparticles functionalized by the nitrogen containing ligands would contribute to the excellent CO_2_ conversion performance. This work would provide valuable insights and opportunities for the rational design of Cu-based MOF catalysts for highly efficient conversion of CO_2_ towards hydrocarbon generation.

## Conflicts of interest

There are no conflicts to declare.

## Supplementary Material

Supplementary information
