# Supplementary material for: Highly efficient electroconversion of carbon dioxide into hydrocarbons by cathodized copper–organic frameworks
Source: Chem Sci. 2019 Jul 2;10(34):7975–81. doi: 10.1039/c9sc02605c (PMC6839807; doi:10.1039/c9sc02605c)
Supplement: Supplementary file 1 [file SC-010-C9SC02605C-s001.pdf]

Electronic Supplementary Material (ESI) for Chemical Science.
This journal is © The Royal Society of Chemistry 2019

Electronic Supplementary Information

Highly Efficient Electroconversion of Carbon Dioxide into Hydrocarbons by Cathodized Copper-organic Frameworks

*Fan Yang, Aling Chen, Pei Lin Deng, Yinzheng Zhou, Zaman Shahid, Hongfang Liu, and Bao Yu Xia**

Key Laboratory of Material Chemistry for Energy Conversion and Storage (Ministry of Education), Key Laboratory of Material Chemistry and Service Failure, School of Chemistry and Chemical Engineering, Wuhan National Laboratory for Optoelectronics, Huazhong University of Science and Technology (HUST), 1037 Luoyu Road, Wuhan 430074, PR China

Corresponding authors: byxia@hust.edu.cn (B.Y. Xia)

Materials

Methanol, acetic acid, copper(ii) nitrate hydrate $Cu(NO_3)_2 \cdot 3H_2O$, and adenine were purchased from Sinopharm Chemical Reagent Co. Ltd. All chemicals were used without further purification. Nafion N-117 membrane was purchased from Dupont. The ultrapure water was obtained from milli-Q system. High purity argon gas (Ar, 99.9999%) and carbon dioxide (CO_2, 99.999%) were purchased from Hua er wen Gas ltd.

Preparation of electrocatalysts

1 mmol acetic acid injected in 20 mL methanol/water solution and the methanol and water were 10 mL each. It was dropwise added into a solution of $Cu(NO_3)_2 \cdot 3H_2O$ (1 mmol) and adenine (1 mmol) in 60 ml methanol/water under vigorous magnetic stirring one day at room temperature. Tuning the volume ratio of methanol and water, the morphologies of Cu-ade MOF changed from the bulk to nanosheet structure. The methanol was 60 mL for Cu-ade nanoplate, the methanol and water were 50 mL and 10 mL respectively for Cu-ade nanocuboid, in addition, the methanol and water were 40 mL and 20 mL respectively for Cu-ade nanosheet. The products washed by ethanol several times and collected by centrifugation. The final purple materials dried in oven box at 60 °C. The Cu-ade nanosheet MOF was annealed at 300 ^oC for 1 hour under air with 1 ^oC/min to obtain the CuO nanosheet material.

Characterizations

The crystalline structures of the Cu-ade MOF were identified by X-ray diffraction (XRD), which were conducted on x'pert3 powder diffractometer equipped Cu Kα (λ = 1.54056 Å) radiation (60kV, 60 mA), the data were analyzed with JADE. The SEM images characterized the morphology and were

performed on Sirion 200 microscopy. The transmission electron microscopy (TEM) images, high-resolution TEM (HRTEM) images, selected area electron diffraction (SAED) patterns and energy-dispersive X-ray (EDX) patterns were obtained by using Talos F200X microscopy (200 KV). The X-ray photoelectron spectroscopy (XPS) spectras were collected by AXIS-ULTRA DLD-600W spectrometer with the dual-chromatized Al and Mg as excitation sources and investigated the change of Cu valence state. And all the binding energies were calibrated by C 1s peak (284.6 eV). The Fourier-transform infrared spectroscopy (FT-IR) pattern was performed on VERTEX 70 to investigate the organic functional groups, the wavenumber ranged from 400 to 4000 cm^{-1}.

Electrochemical measurements and products analysis

5 mg catalyst was dispersed into 500 μL ethanol and 25 μL Nafion solution (5 wt%), a homogenous catalyst ink was obtained by sonicating for 15 min. 10 μL of the catalyst ink was dropped on to the glass carbon electrode after polished with alumina paste and dried under room temperature. The CO_2 electrochemical reduction experiment were carried out in a sealed H-type cell which separated by Nafion-117 proton exchange membrane. Each cell was injected into 12 mL electrolyte. The working electrode was glassy carbon electrode with catalyst, and Ag/AgCl (saturated KCl) electrode and Platinum net electrode were used as the reference electrode and counter electrode, respectively. The conversion equation: E (vs RHE) = E (vs Ag/AgCl) + 0.21 V + 0.0591 × pH. The electrolyte was 0.1 M potassium bicarbonate ($KHCO_3$) solution. Before the electrochemical tests, CO_2 or Ar gas was bubbled in 0.1 M $KHCO_3$ electrolyte at least 20 min to remove other residual gas, and all tests were carried out at the room temperature (25 °C). The LSV curves of three kinds of Cu-ade catalysts were made with the scan rate of 10 mV s^{-1} in Ar and CO_2 saturated in 0.1 M $KHCO_3$. The cyclic voltammetry measurements were measured from 0 to -0.1 V vs RHE under CO_2 saturated 0.1 M $KHCO_3$ solution

with various scan rates (10, 20, 30, 40, and 50 mV s^{-1}). Electrochemical impedance spectroscopy (EIS) was measured with the frequency range of 0.1 Hz to 100 kHz. R_f=C_{dl} /C_S, in which R_f stands for roughness factor； C_{dl} stands for double layer capacitance, the C_{dl} was determined by measuring the capacitive current associated with double-layer charging from the scan-rate dependence of cyclic voltammetric stripping; C_S stands for the average double-layer capacitance of a smooth metal surface (about 20μF cm^{-2}). ECSA=R_f S, in which R_f stands for roughness factor, S stands for the real surface area of the smooth metal electrode, which was generally equal to the geometric area of glassy carbon electrode (0.196 cm^2).The electrode was applied for constant voltage, the gas products were analyzed by the gas chromatograph (Shimadzu GC-2014) linking to the cathode cell, which was equipped with two flame ionization detectors (FID) and thermal conductivity detector (TCD). The liquid products were inspected by 400 MHz NMR spectrometer.[1]

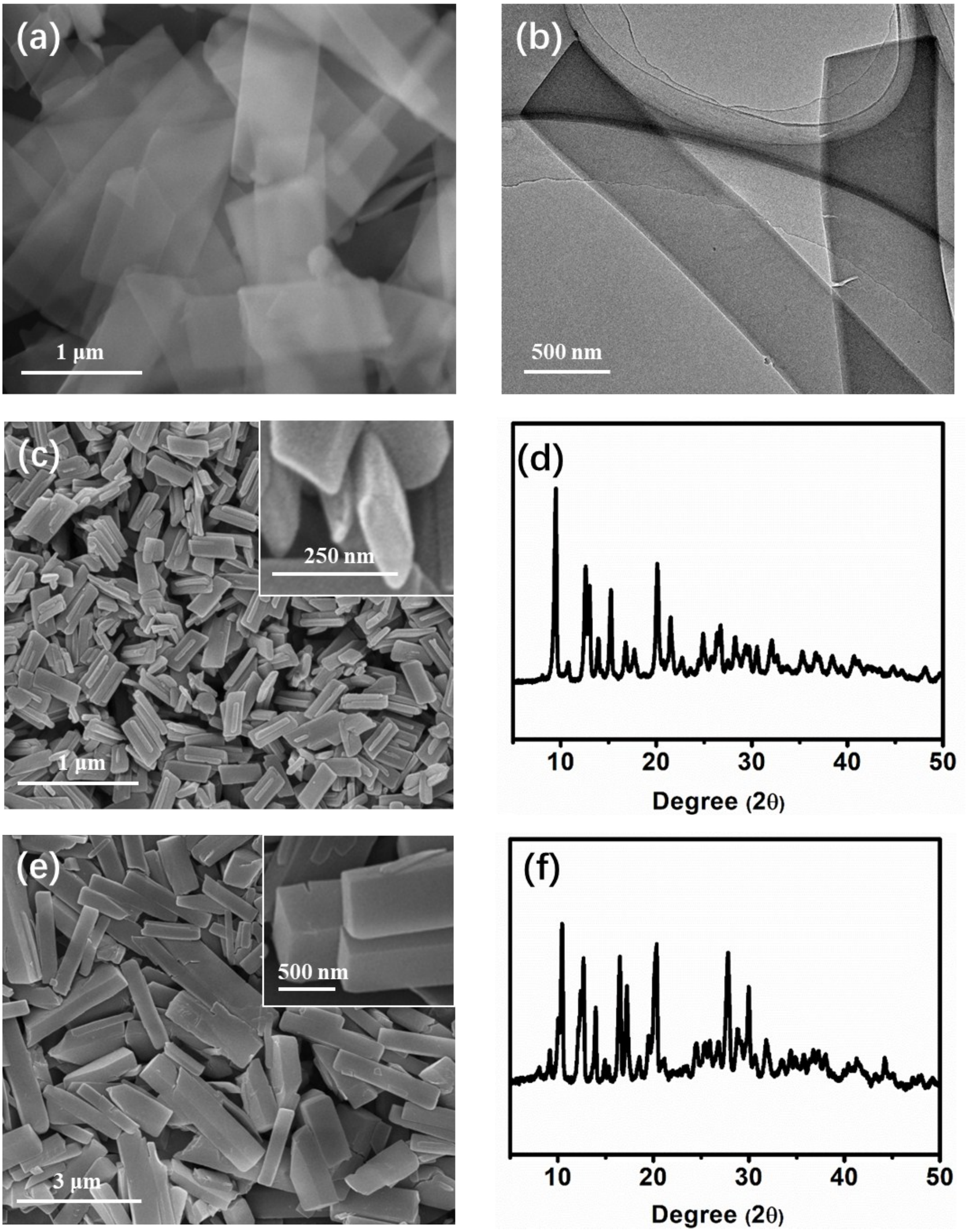

Figure S1. (a) SEM images and (b) TEM images of s-Cu-ade catalyst. (c) SEM images and (d) XRD pattern of p-Cu-ade catalyst. (e) SEM images and (f) XRD pattern of c-Cu-ade catalyst.

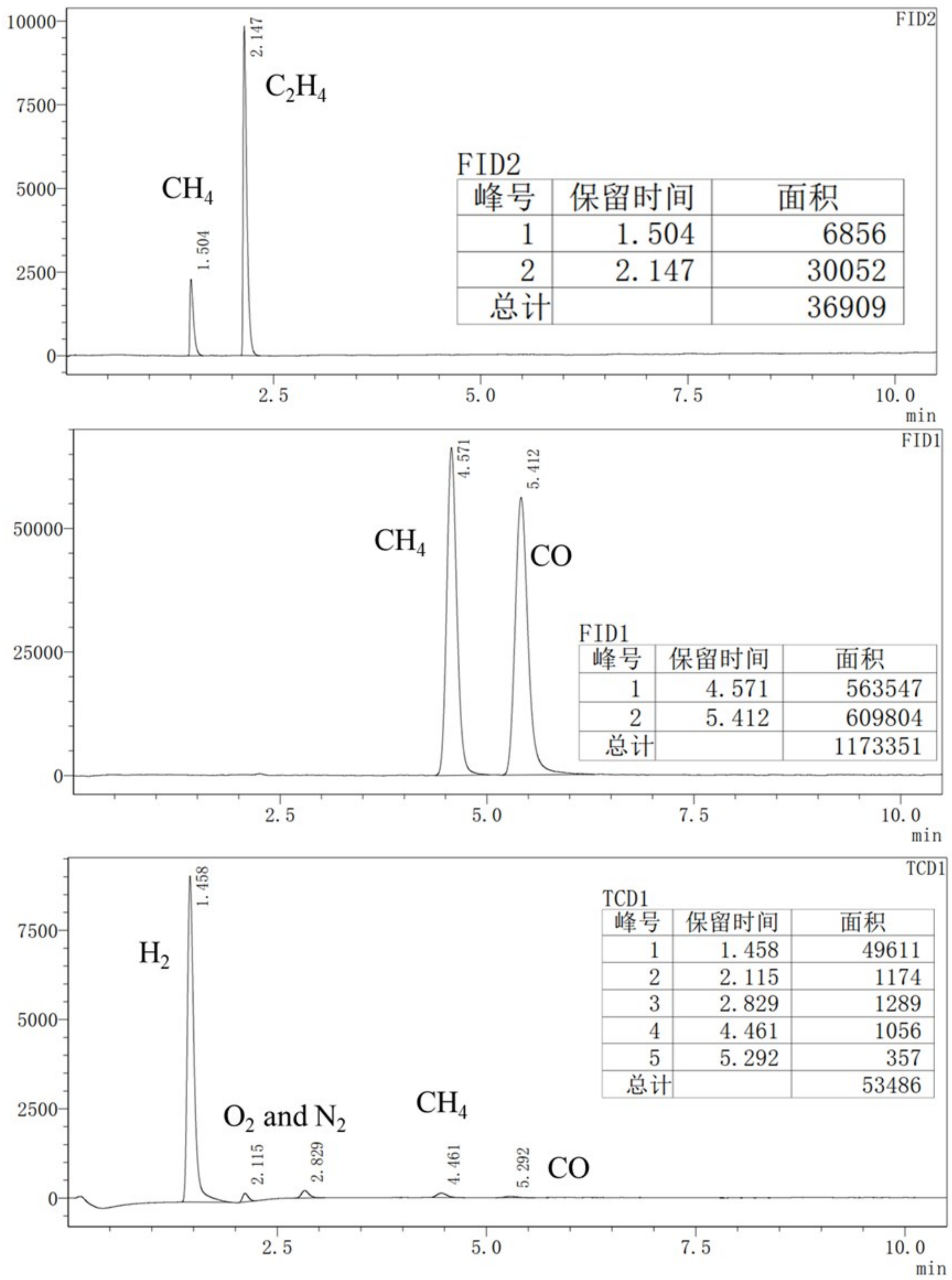

Figure S2. GC measurement of gas products on CO_2 saturated 0.1M $KHCO_3$.

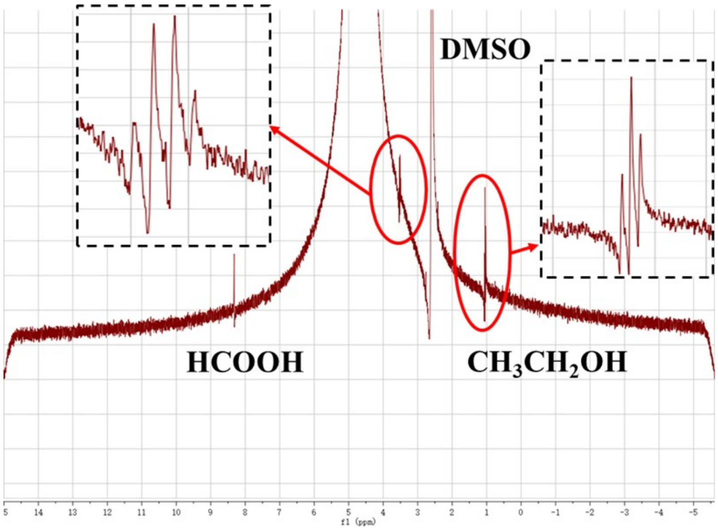

Figure S3. Proton NMR spectrum of liquid products on CO_2 saturated 0.1M $KHCO_3$, internal standard substance (DMSO).

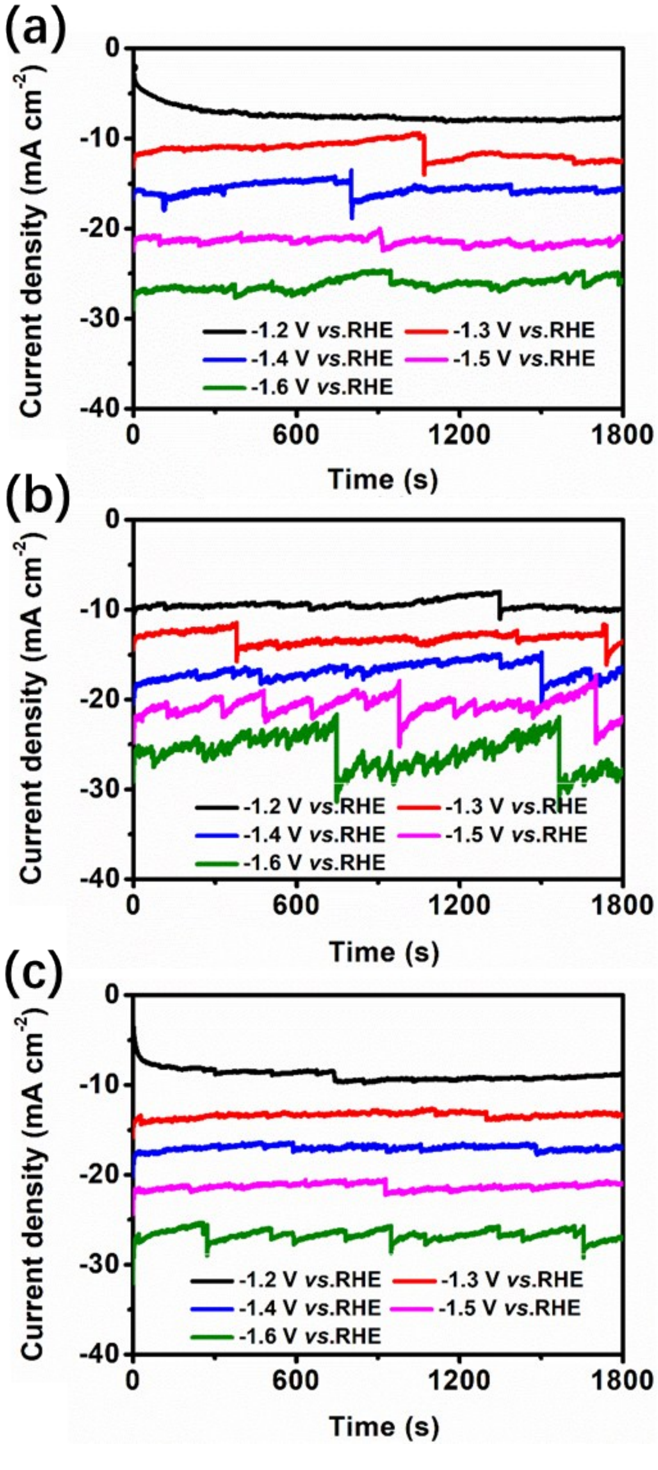

Figure S4. IT curves of different Cu-ade catalysts for CO_2 reduction. (a) p-Cu-ade, (b) c-Cu-ade, (c) s-Cu-ade.

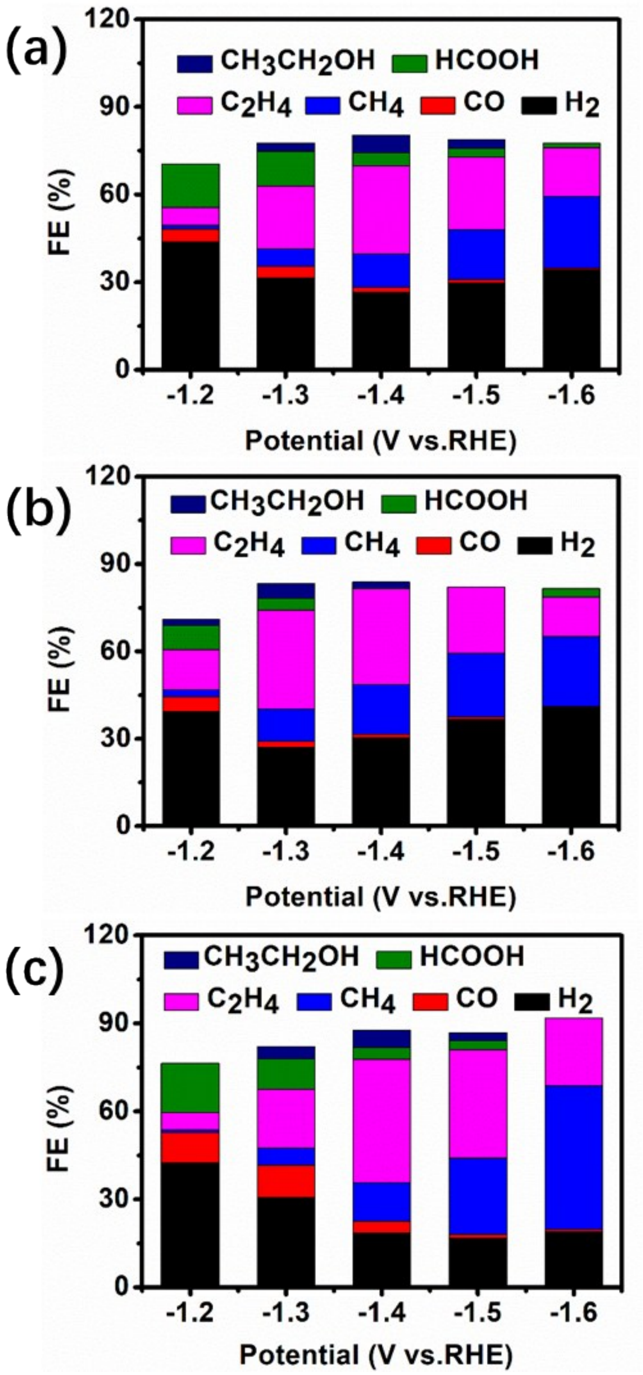

Figure S5. The total FE of different Cu-ade catalysts for CO_2 reduction. (a) p-Cu-ade, (b) c-Cu-ade, (c) s-Cu-ade.

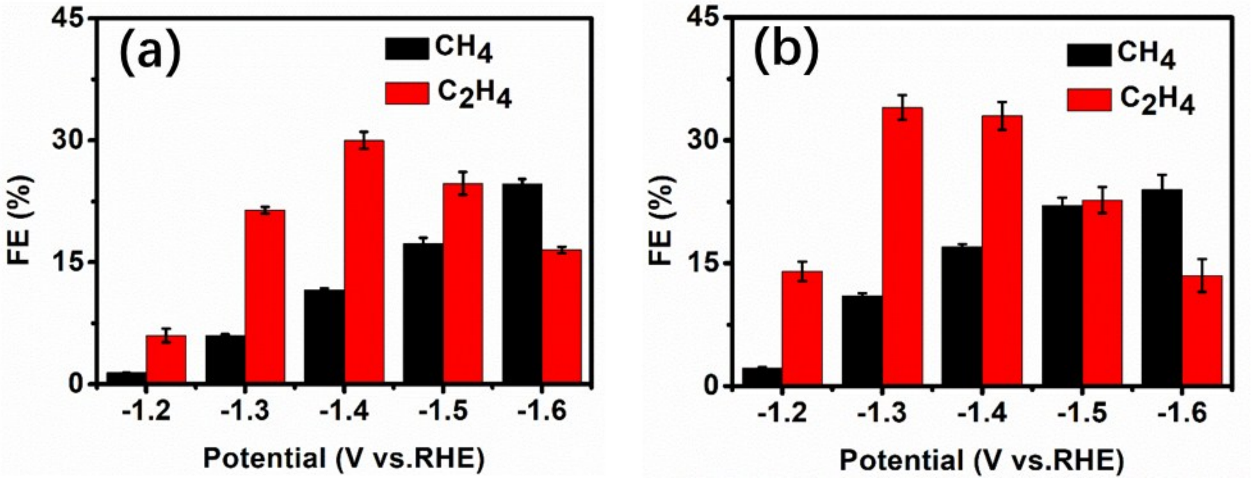

Figure S6. The FE CH_4 and C_2H_4. (a) p-Cu-ade, (b) c-Cu-ade.

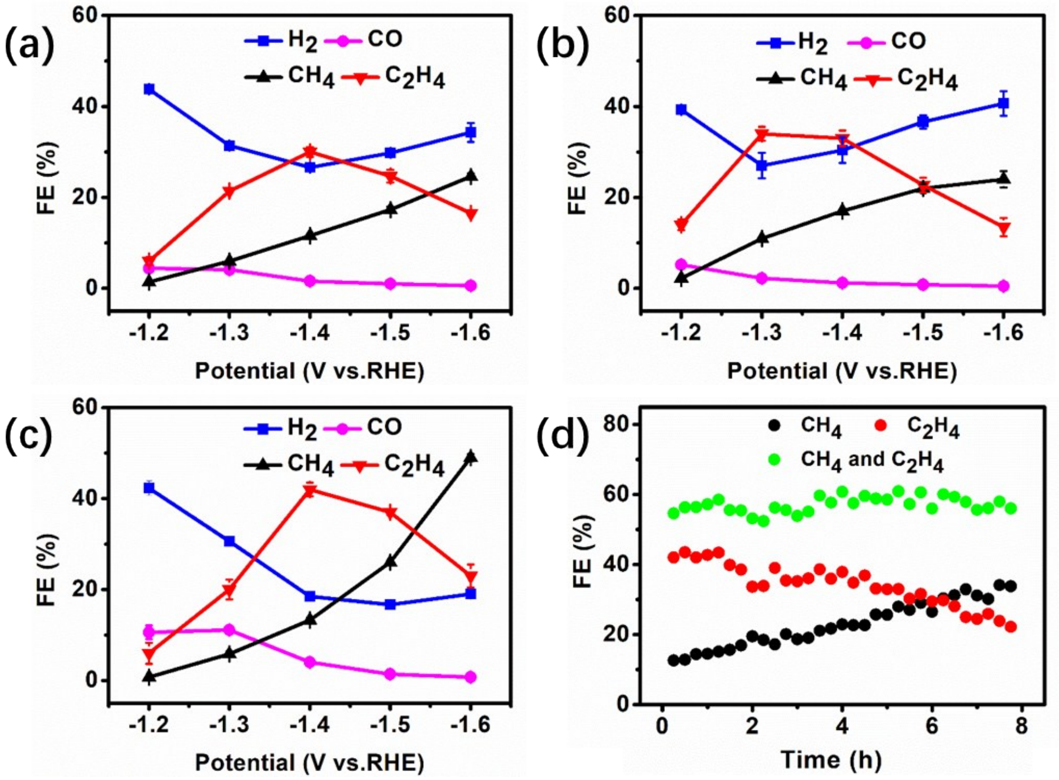

Figure S7. The FE and partial current density of CH_4 and C_2H_4. (a) p-Cu-ade, (b) c-Cu-ade, (c) s-Cu-ade, (d) stability test.

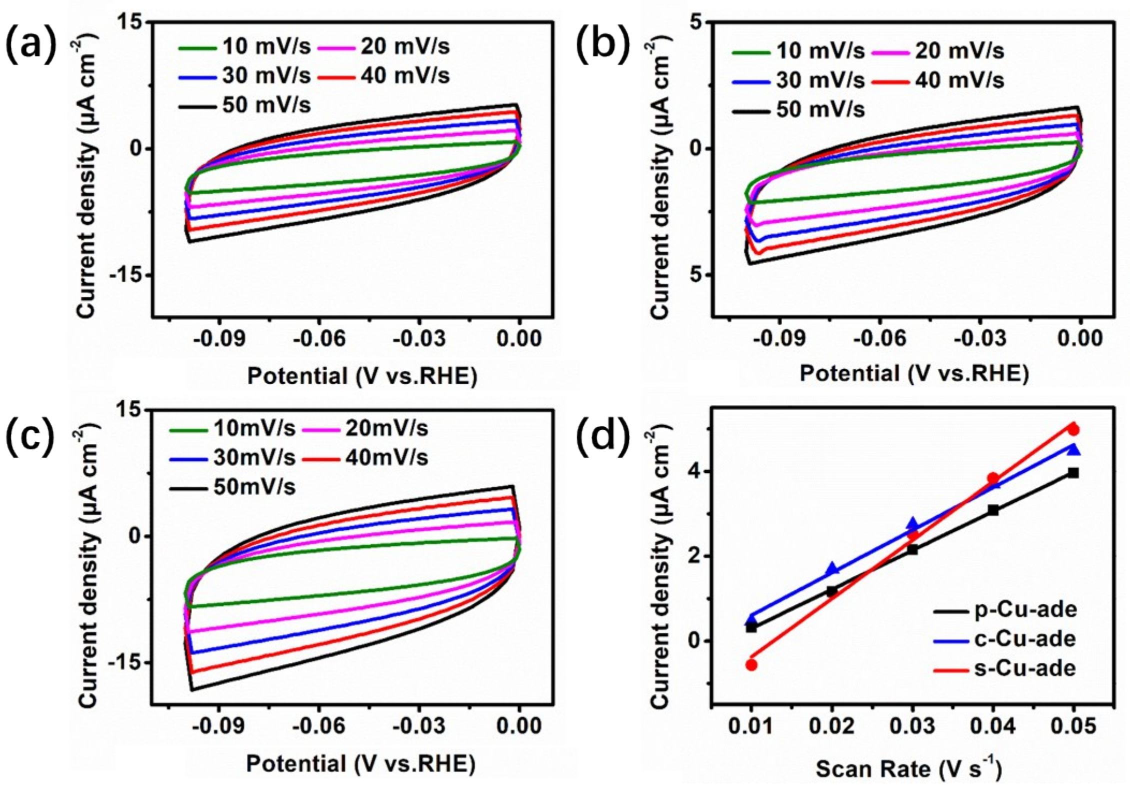

Figure S8. Capacitive behaviors of different Cu-ade catalysts. (a) p-Cu-ade, (b) c-Cu-ade, (c) s-Cu-ade. (d) Plot of the current density with different scan rates range 10 mV/s to 50 mV/s.

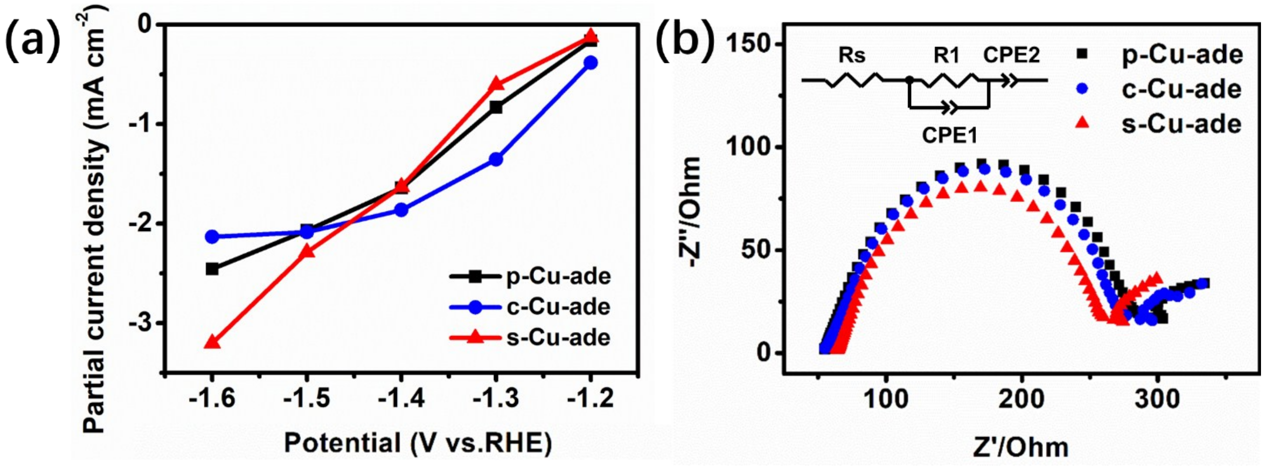

Figure S9. (a) The partial current densities of CH_4 and C_2H_4 over three Cu-ade MOFs corrected by ECSA. (b) Electrochemical impedance spectroscopy (EIS) and the equivalent circuit (insets) of three Cu-ade catalysts.

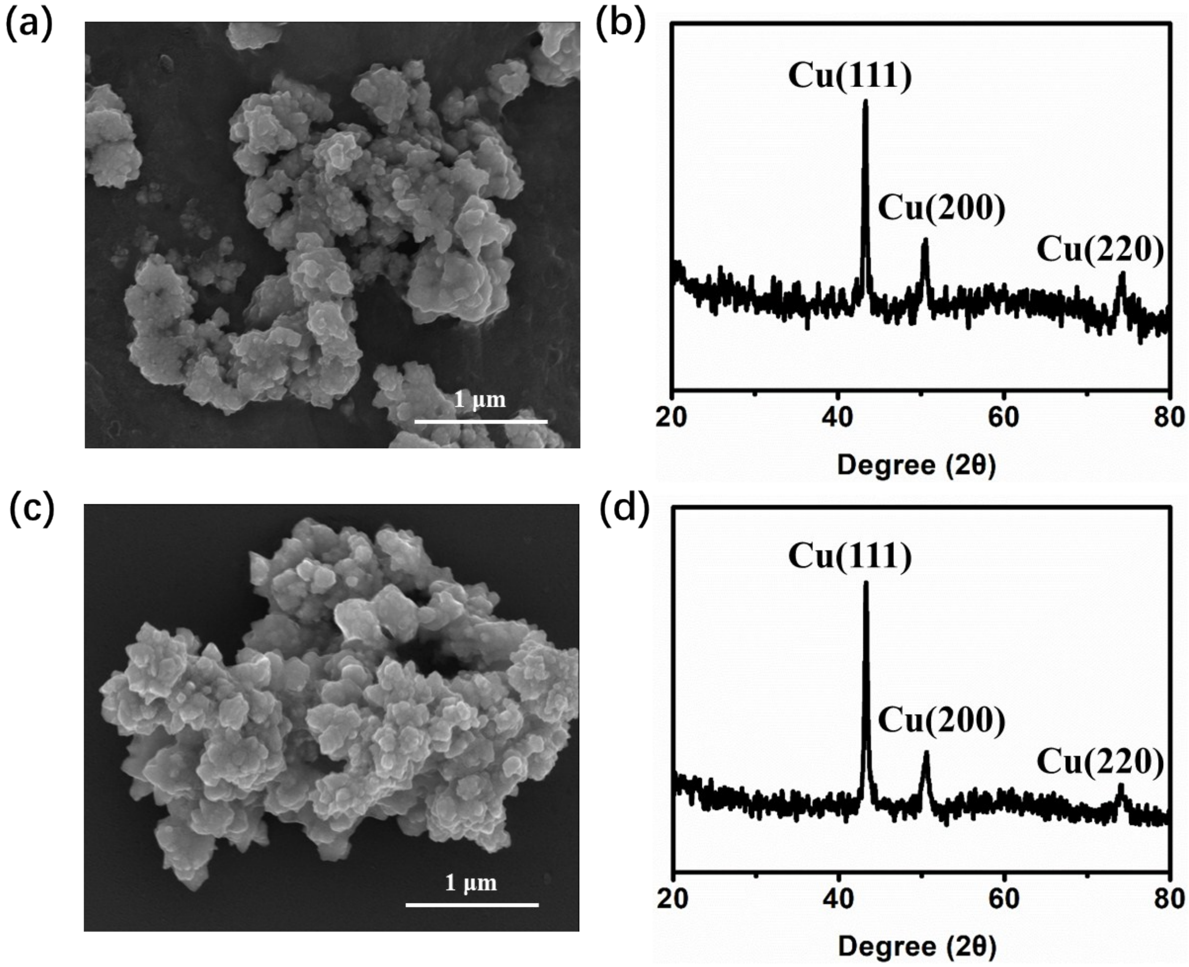

Figure S10. (a) SEM image and (b) XRD pattern of the cathodized p-Cu-ade. (c) SEM image and (d) XRD pattern of the cathodized c-Cu-ade.

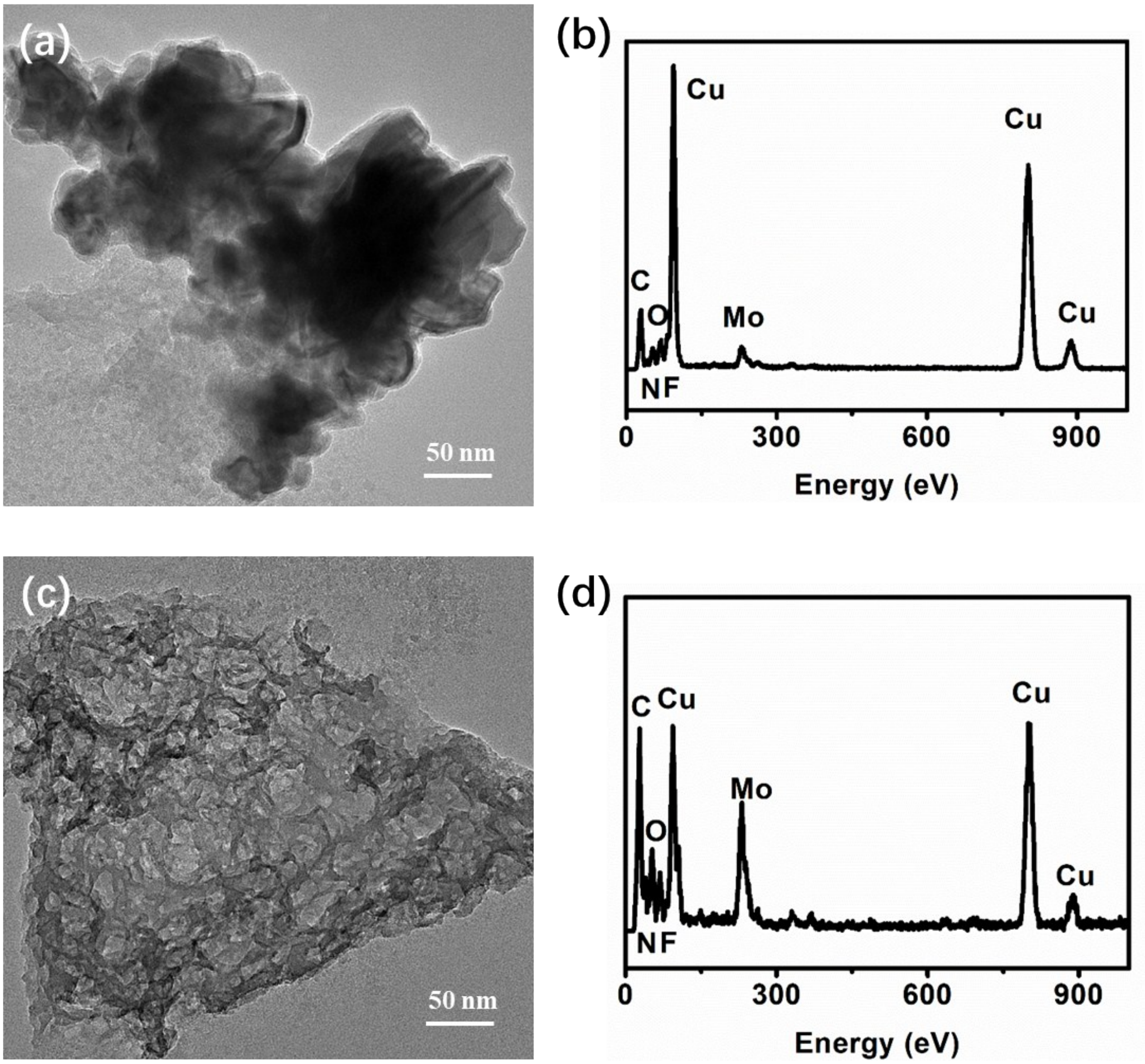

Figure S11. (a) Copper enrichment region and (c) Carbon enrichment region TEM image of the cathodized s-Cu-ade catalysts. (b) and (d) the EDS spectroscopy of the (a) and (c), respectively. Mo is background elements due to the Mo Micro grid, F element belongs to Nafion solution.

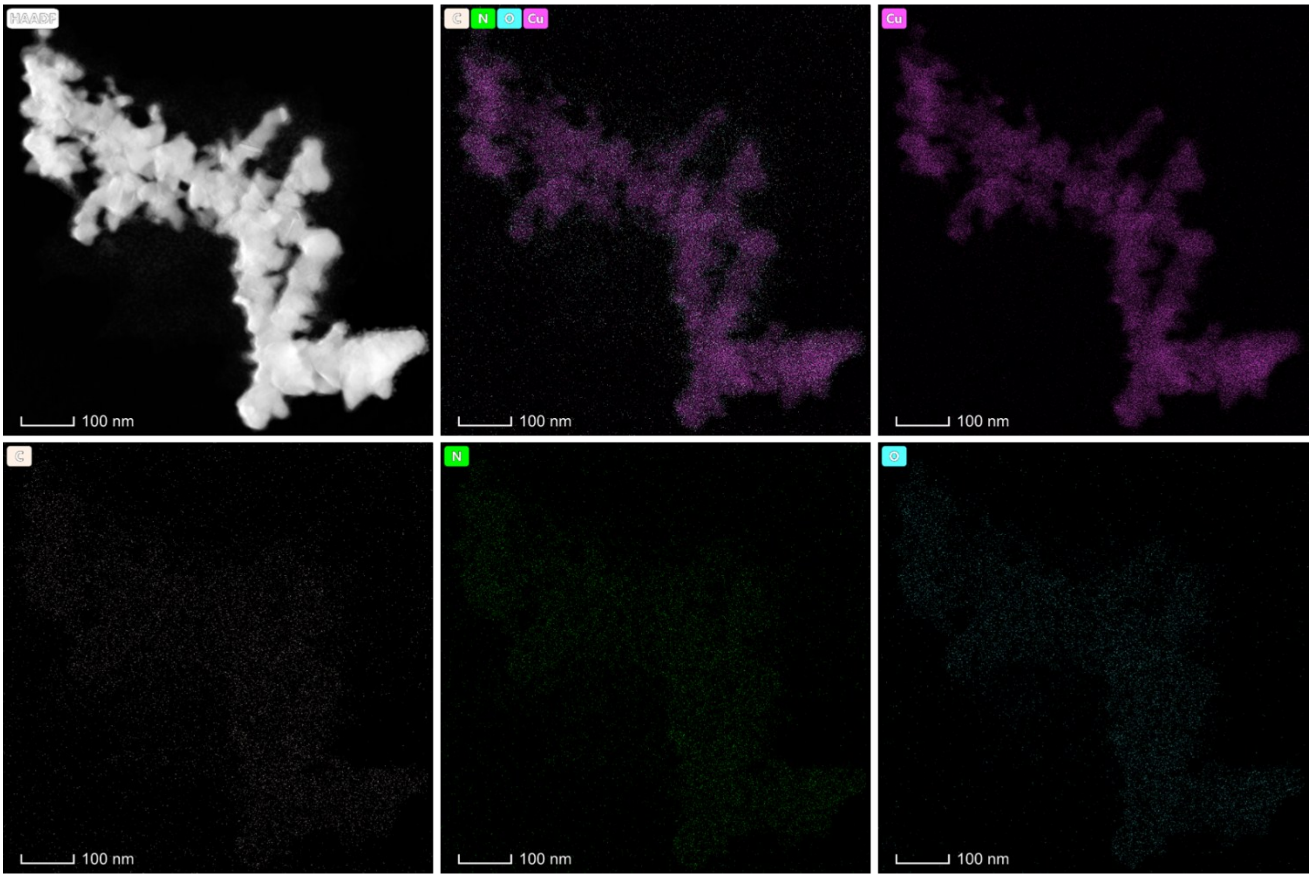

Figure S12. High-angle annular dark field-scanning transmitted electron images (HAADF-STEM) and STEM-EDS elemental maps of the cathodized s-Cu-ade.

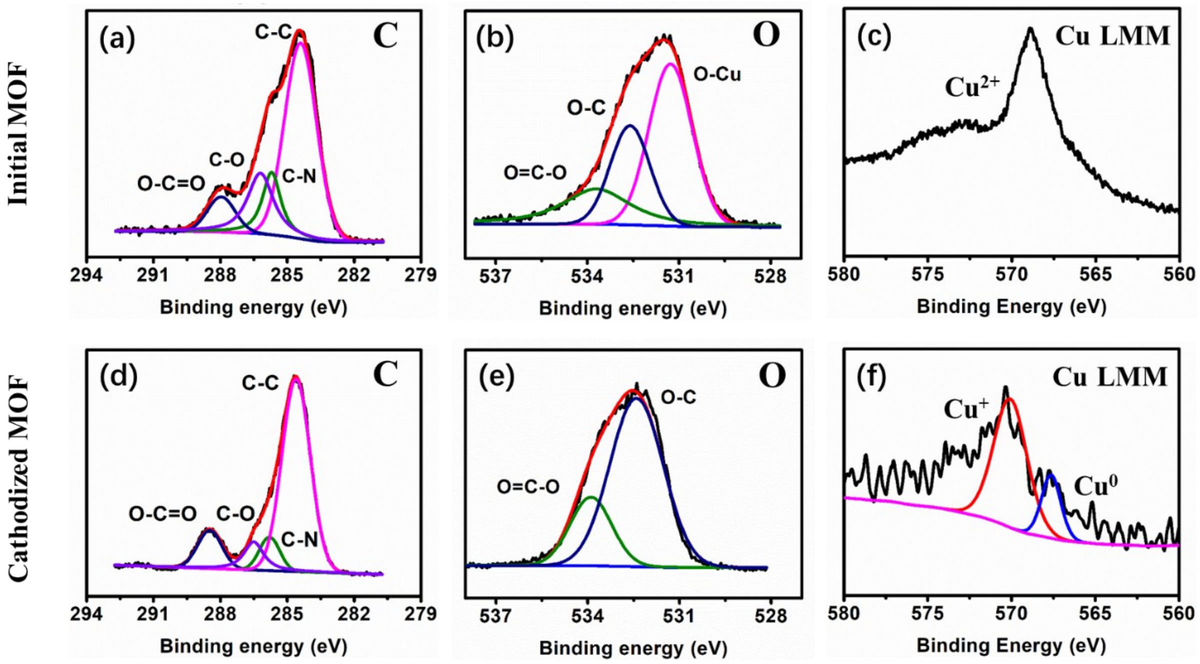

Figure S13. XPS patterns of the initial and cathodized s-Cu-ade MOF. (a) and (d) C1s scan XPS patterns, (b) and (e) O1s scan XPS patterns, (c) and (f) Cu LMM scan XPS patterns.

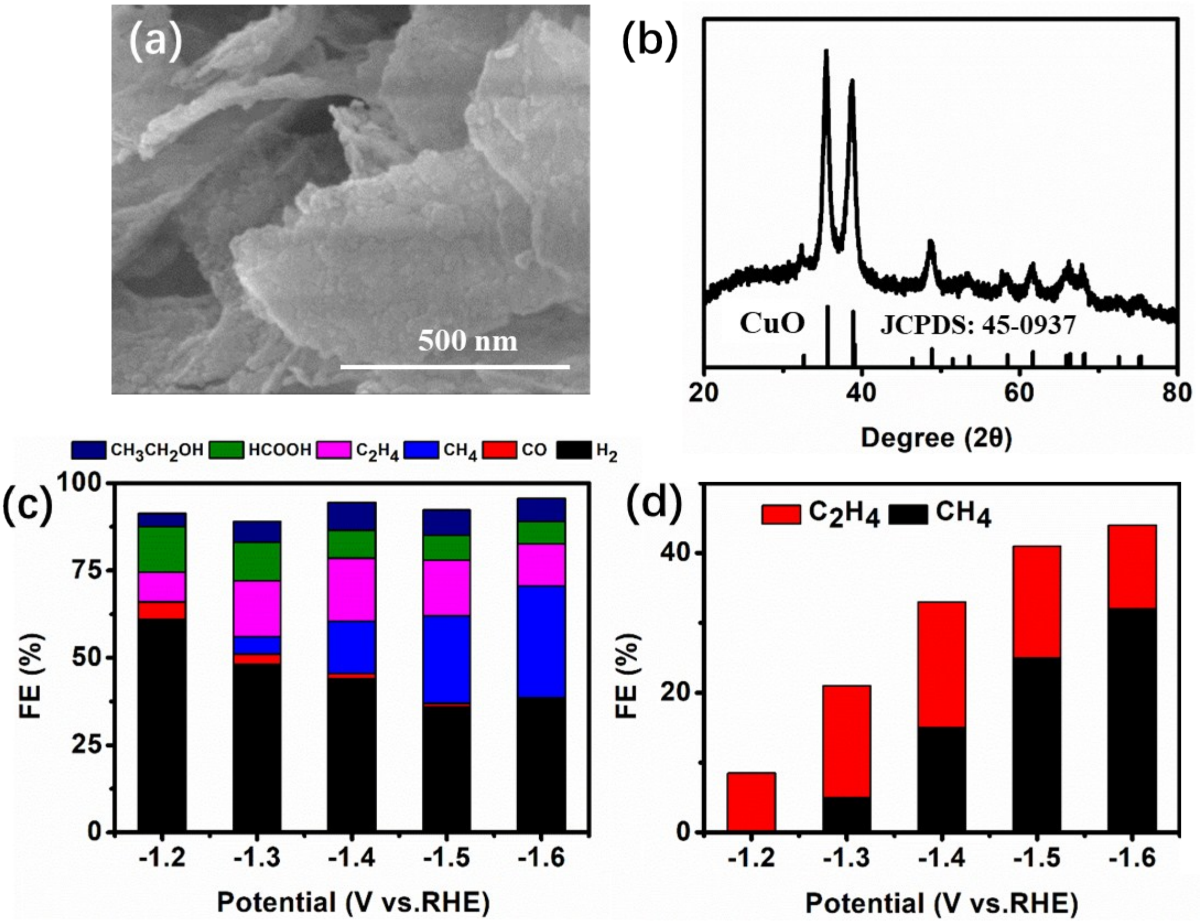

Figure S14. (a) SEM image, (b) XRD pattern, (c) Total FE, (d) FE of CH_4 and C_2H_4 for CuO nanosheet.

Table S1. Recent Cu-organic complex for CO_2 electrocatalysis.

Catalyst	Organic ligand	FE (HCOOH)	FE (CH_4)	FE (C_2H_4)	FE (CH_3COOH)	Ref
porphyrin–Cu(II) complex	porphyrin	68.4%			~15%	[2]
Cu porphyrin molecules	porphyrin		~30%	~10%		[3]
Cu phthalocyanine	phthalocyanine		66%			[4]
HKUST-1	Benzene-1,3,5-tricarboxylate		27%			[4]
Cu nanoparticles installed in NU-1000	H_4TBAPy	~30%				[5]
Cu-based MOF-74	2,5-dihydroxyterephthalic acid		~50%			[6]
$Cu_3(BTC)_2$	1,3,5-tricarboxylic acid		~35%	~12%		[7]
**Cu-ade**	**adenine and acetic acid**		**~12%**	**45%**		**This work**

Reference

[1] S. Gao, Y. Lin, X. Jiao, Y. Sun, Q. Luo, W. Zhang, D. Li, J. Yang, Y. Xie, *Nature* **2016**, *529*, 68.
[2] J.-X. Wu, S.-Z. Hou, X.-D. Zhang, M. Xu, H.-F. Yang, P.-S. Cao, Z.-Y. Gu, *Chem. Sci.* **2019**, *10*, 2199.
[3] Z. Weng, J. Jiang, Y. Wu, Z. Wu, X. Guo, K. L. Materna, W. Liu, V. S. Batista, G. W. Brudvig, H. Wang, *J. Am. Chem. Soc.* **2016**, *138*, 8076.
[4] Z. Weng, Y. Wu, M. Wang, J. Jiang, K. Yang, S. Huo, X.-F. Wang, Q. Ma, G. W. Brudvig, V. S. Batista, Y. Liang, Z. Feng, H. Wang, *Nat. Commun.* **2018**, *9*, 415.
[5] C.-W. Kung, C. O. Audu, A. W. Peters, H. Noh, O. K. Farha, J. T. Hupp, *ACS Energy Lett.* **2017**, *2*, 2394.
[6] M. K. Kim, H. J. Kim, H. Lim, Y. Kwon, H. M. Jeong, *Electrochimica Acta* **2019**.
[7] X. Tan, C. Yu, C. Zhao, H. Huang, X. Yao, X. Han, W. Guo, S. Cui, H. Huang, J. Qiu, *ACS Appl. Mater. Inter.* **2019**.
